# Determination of the Sediment Carrying Capacity Based on Perturbed Theory

**DOI:** 10.1155/2014/240858

**Published:** 2014-07-17

**Authors:** Zhi-hui Ni, Qiang Zeng, Wu Li-chun

**Affiliations:** ^1^Key Laboratory of Hydraulic and Waterway Engineering of the Ministry of Education and National Engineering Research Center for Inland Waterway Regulation, Chongqing Jiaotong University, Chongqing 400074, China; ^2^Southwestern Research Institute of Water Transportation Engineering, Chongqing Jiaotong University, Chongqing 400016, China; ^3^Chongqing Education University, Chongqing 400067, China

## Abstract

According to the previous studies of sediment carrying capacity, a new method of sediment carrying capacity on perturbed theory was proposed. By taking into account the average water depth, average flow velocity, settling velocity, and other influencing factors and introducing the median grain size as one main influencing factor in deriving the new formula, we established a new sediment carrying capacity formula. The coefficients were determined by the principle of dimensional analysis, multiple linear regression method, and the least square method. After that, the new formula was verified through measuring data of natural rivers and flume tests and comparing the verified results calculated by Cao Formula, Zhang Formula, Li Formula, Engelung-Hansen Formula, Ackers-White Formula, and Yang Formula. According to the compared results, it can be seen that the new method is of high accuracy. It could be a useful reference for the determination of sediment carrying capacity.

## 1. Introduction

Sediment carrying capacity reflects the account of entrainment and transportation by the flow under the certain boundary condition. It is a comprehensive index characterizing the sediment carrying capacity of flow under the conditions of equilibrium of scouring and deposition. For a long time, the sediment carrying capacity is an important issue in the field of sediment study; it involves sediment transport, pollutant diffusion, evolution of beach, sea bed erosion, and many other issues.

For half a century, scholars at home and abroad had done a lot of studies on the sediment carrying capacity and got a series of results [1–4]. At present, the more common sediment carrying capacity formula is obtained by Zhang [5] because of its harmony dimension, clear mechanical mechanism, and well expressed contradictory relationship between turbulence and gravity effects. Zhang Formula is derived by the measured data of Yangtze River, Yellow River, channels, and flume tests based on the hypothesis of turbulence restriction. But in practical engineering applications, it is difficult to find a unified *K* and *m* value. Yang [6] established a new sediment carrying capacity formula including the sandy bed load, and this formula is based on the research of unit stream power by theoretical models. Also, Yang made detailed analyses and comparisons between Velikanov's gravitational power theory [7] and his unit stream power theory from theoretical point of view using field and laboratory data for verification in “*Sediment Transport Theory and Practice*” [8]. By considering the bed load transport rate and combining it with the law of suspended sediment along the vertical distribution, Wang [9] obtained the sediment carrying capacity formula of suspended sediment. Based on the theory of sediment movement and energy balance, Xing et al. [10] derived a half-tide average sediment carrying capacity formula at Yangtze river Delta that combined the researches of Deng's and Dou's. There are so many sediment carrying capacity formulas like the ones above [11, 12]. From the above analysis, it is easy to find that most of the sediment carrying capacity formulas have adopted two common hydraulic factors (velocity and depth) and sediment factor (settling velocity), and the difference is the index of each factor.

By considering the impact of sediment grain size, Mai and Zhao [13] proposed a sediment carrying capacity formula based on the measured data of Yellow River. Chien and Wan [14] summarized and analyzed the Einstein Formula, Bagnold Formula, Engelund-Hansen Formula, Ackers-White Formula, Zhang Formula, Sha formula, and other formulas and suggested that the relative roughness is a main influencing factor in deriving the sediment carrying capacity in “*Mechanics of Sediment Transport*.”

The perturbed theory started from 1882. It is closely united to fluid mechanics and other mechanics disciplines. Ni and Zhou [15] obtained the law of suspended sediment concentration by momentum equation based on the perturbed theory.

Internationally, there are so many achievements on sediment carrying capacity. Engelund-Hansen Formula, Ackers-White Formula, and Yang Formula are commoner ones. Engelund and Hansen [16] assumed that the mean velocity of sediment is proportional to friction velocity and obtained a new sediment carrying capacity formula in the stage of dune and sand waves by introducing flow intensity parameter Θ and sediment transport intensity parameter Φ_*T*_, *f*Φ_*T*_ = 0.4Θ^5/2^, Φ_*T*_ = (*g*_*T*_/*γ*_*s*_)(*γ*/(*γ*_*s*_ − *γ*))^1/2^(1/*gD* ^3^)^1/2^, Θ = (*γ*/(*γ*_*s*_ − *γ*))(*U*_∗_^2^/*gD*), where *f* is the Darcy coefficient, Φ_*T*_ is sediment transport intensity parameter, Θ is flow intensity parameter, and *g*_*T*_ is single-wide transport rate of total sediment. Different to Engelund and Hansen, Ackers and White [17] assumed that the efficiency of sediment carrying by the flow is related to sediment movement intensity, effective tractive force, and weight of surface sediment particles in the water, and then they verified their conjecture by a large number of measured data. At last, they derived a new sediment carrying capacity formula based on the flume tests, *Y* = *c*((*M*/*A*) − 1)^*m*^, $M=(U_{*}^{n}/\sqrt{gD(\left(\gamma_{s}-\gamma \right)/\gamma )}){\left[U/\sqrt{32}\log(10h/D)\right]}^{1-n}$, where *Y* is sediment transport parameter, *M* is flow intensity parameter, *c*, *A*, *m*, and *n* are fitting parameters, *U*_∗_ is friction velocity, *U* is mean velocity of each profile, *D* is sediment grain size, *γ*_*s*_, *γ* are bulk densities of sediment and water, and *h* is water depth. Thinking of the theoretical model of unit stream power, Yang [6] derived a new bed load sediment carrying capacity formula, lg*S*_∗_ = *a*_1_ + *a*_2_lg⁡(*ωD*/*U*) + *a*_3_lg⁡(*U*_∗_/*ω*)+[*b*_1_ + *b*_2_lg⁡(*ωD*/*U*) + *b*_3_lg⁡(*U*_∗_/*ω*)] · lg((*UJ*/*ω*)−(*U*_*c*_ *J*/*ω*)), where *S*_∗_ is sediment carrying capacity, *ω* is the settling velocity, *U*_*c*_ is starting velocity, and *a*_1_, *a*_2_, *a*_3_, *b*_1_, *b*_2_, and *b*_3_ are the fitting parameters.

Also, some famous sediment carrying capacity formulas had been derived in China. According to the hypothesis of turbulence restriction, Zhang [5] established the energy balance equations and then derived the formula (*S*_∗_ = *K*(*U* ^3^/*gRω*)^*m*^) based on the principle of energy balance, where *K* is the coefficient including dimension (kg/m^3^), *m* is the index, *U* is the average velocity (m/s), *g* is the gravity acceleration (m/s^2^), *h* is the water depth (m), and *ω* is the settling velocity (mm/s). Cao et al. [18] improved the Zhang Formula according to the balance of saturated sediment data, and he obtained a new sediment carrying capacity formula (*S*_∗_ = 0.385(*γ*_*m*_/(*γ*_*s*_ − *γ*_*m*_))(*U* ^3^/*gRω*_*ms*_)), where *γ*_*m*_ is bulk density of sediment (g/cm^3^), *γ*_*s*_ is the bulk density of muddy water (g/cm^3^), *R* is the hydraulic radius (m), and *ω*_*ms*_ is the corresponding settling velocity (mm/s). Li [19] thought that there is certain linear relationship between and relative roughness and proposed a new sediment carrying capacity formula (*S*_∗_ = *K*_0_(0.1 + 90(*ω*/*U*))(*U* ^3^/*ghω*)^*m*^) based on it, where *K*_0_ = 0.245, *m* = 1.0, and *U* is average velocity of water vertical line (m/s).

The paper will analyze the major factors by using perturbed theory and then derive a new sediment carrying capacity formula. After that, the measured data of home and abroad rivers will be used to verify and analyze the new formula. Simultaneously, some tank tests by famous scholars should be used for further validation.

## 2. Methodology Description

### 2.1. Introduction of Perturbed Theory

Perturbed theory gradually developed in solving nonlinear equations with small parameter. It differs from conventional ones because the main task of the evolutionary method established on the perturbed theory is to solve the series of small parameter in expansion. Then the original problem is transformed to gradually solve the coefficient of the expansion. In this way, the value will be close to the true value, and it is easier to other iterative methods and always the reason can be solved.

When using perturbed theory to solve the problem, firstly we must utilize dimensionless method to analyze the equation and then propose the perturbed variable. For example, we utilized dimensionless method to analyze the influencing factor *p*, and at the same time, we proposed the perturbed variable *ε*; then we adopted perturbed theory to analyze the influencing factor *p*; after that we have

(1) $$\begin{array}{c} p=p_{0}+\varepsilon p_{1}+\varepsilon^{2}p_{2}+\cdots . \end{array}$$

Due to the gradualness of asymptotic series, usually taking a few can get very high precision and sometimes even reach the exact solution. At present, we usually take the first-order or second-order type of the analytic expression.

### 2.2. Derivation of the New Equation

The factors influencing the sediment carrying capacity included velocity (*U*), water depth (*h*), gradient ratio (*J*), gravity action result in flow (*g*), viscosity (*ν*), effective bulk density (*γ*_*s*_ − *γ*), settling velocity (*ω*), median grain size (*D*_50_), and composition of the river bed and river width (*B*). Therefore, the sediment carrying capacity formula can be written as a function of the following form:

(2) $$\begin{array}{c} S_{*}=f\left(U,h,J,g,\nu ,\gamma_{s}-\gamma ,w,D_{50},B\right). \end{array}$$

Taking *U*, *h*, and *γ* as the basic variables, then (2) can be written as a dimensionless formula:

(3) $$\begin{array}{c} S_{*}=f\left(\frac{U^{2}}{gh},\frac{\gamma_{s}-\gamma }{\gamma },\frac{Uh}{\nu },\frac{U}{\omega },\frac{D_{50}}{h},\frac{B}{h}\right). \end{array}$$

For the natural river, (*γ*_*s*_ − *γ*)/*γ* is a constant, the effects of flow viscosity and the side walls. Therefore, (3) can be converted into

(4) $$\begin{array}{c} S_{*}=f\left(\frac{U^{2}}{gh},\frac{U}{\omega },\frac{D_{50}}{h}\right). \end{array}$$

According to the home and abroad researches it can be found that, in fact, the basic expression of most experienced and semiempirical formulas derived from natural rivers is (4). This paper will analyze by perturbed theory combining the contrast relation between turbulence intensity of the flow (*U* ^2^/*gh*) and gravity action (*U*/*ω*) with the contrast relation between turbulence intensity of the flow (*U* ^2^/*gh*) and relative roughness (*D*_50_/*h*).

Analyzing the contrast relation between turbulence intensity of flow (*U* ^2^/*gh*) and the relative roughness (*D*_50_/*h*) on perturbed theory, we get

(5) $$\begin{array}{c} S_{*1}=S_{0}+\varepsilon_{1}\frac{U^{2}D_{50}}{gh^{2}}+\varepsilon_{1}^{2}\frac{U^{2}D_{50}}{gh^{2}}+\varepsilon_{1}^{3}\frac{U^{2}D_{50}}{gh^{2}}+\cdots , \end{array}$$

where *ε*_1_ is the perturbation variable.

Analyzing the contrast relation between turbulence intensity of flow (*U* ^2^/*gh*) and gravity action (*U*/*ω*) on perturbed theory, we get

(6) $$\begin{array}{c} {S_{*}}_{2}=S_{1}+\varepsilon_{2}\frac{U^{3}}{gh\omega }+\varepsilon_{2}^{2}\frac{U^{3}}{gh\omega }+\varepsilon_{2}^{3}\frac{U^{3}}{gh\omega }+\cdots , \end{array}$$

where *ε*_2_ is the perturbation variable.

Equations (5) and (6) can be transformed into

(7) S∗1=S0+ε1U2D50gh2+ε12U2D50gh2,S∗2=S1+ε2U3ghω+ε22U3ghω,

where  *ε*_1_ and *ε*_2_ are small values; therefore, *ε*_1_^2^ and *ε*_2_^2^ are smaller. Because of that, the values of *ε*_1_^2^(*U* ^2^ *D*_50_/*gh* ^2^) and *ε*_2_^2^(*U* ^3^/*ghω*) could be neglected. Combining the above expressions, (7) can be written as follows:

(8) $$\begin{array}{c} S_{*}={S_{*}}_{1}+S_{*2}=S_{0}+S_{1}+\varepsilon_{1}\frac{U^{2}D_{50}}{gh^{2}}+\varepsilon_{2}\frac{U^{3}}{gh\omega }. \end{array}$$

To make (8) more intuitive, we use *k*_1_ replacing *ε*_1_, *k*_2_ replacing *ε*_2_, and *k*_3_ replacing *S*_0_ + *S*_1_; then we can get

(9) $$\begin{array}{c} S_{*}=k_{1}\frac{U^{2}D_{50}}{gh^{2}}+k_{2}\frac{U^{3}}{gh\omega }+k_{3}. \end{array}$$

### 2.3. Determination of Physical Parameters

According to Ni et al. [36–38], the least square method combination of enumeration can be used to determine the coefficients. Concrete steps are as follows.

(1) The establishment of objective function
  (10) $$\begin{array}{c} f={\overset{n}{\underset{i=1}{\sum }}\left(k_{1}\frac{U_{i}^{2}D_{50i}}{gh_{i}^{2}}+k_{2}\frac{U_{i}^{3}}{gh_{i}\omega_{i}}+k_{3}-{S_{*}}_{i}\right)}^{2}, \end{array}$$
 where *U*_*i*_, *D*_50*i*_, *h*_*i*_, *ω*_*i*_, and *S*_∗__*i*_ represent the *i*th layer flow velocity, median grain size, water depth, settling velocity, and sediment carrying capacity. *n* is the total number of the layers considered.
(2) When the objective function reaches the minimum, the following conditions are satisfied:
  (11) ∂f∂k1=0⟹2∑i=1nUi2D50ighi2(k1Ui2D50ighi2+k2Ui3ghiωi+k3−S∗i)=0,∂f∂k2=0⟹2∑i=1nUi3ghiωi(k1Ui2D50ighi2+k2Ui3ghiωi+k3−S∗i)=0,∂f∂k3=0⟹2∑i=1n(k1Ui2D50ighi2+k2Ui3ghiωi+k3−S∗i)=0,
 where *n* is the total number of the layers for fitting. Solving (11) with the matrix method gives *k*_1_, *k*_2_, and *k*_3_.

Analyzing 1633 domestic and foreign measured data by multivariate linear regression analysis combination of the least square method, the values of coefficients can be obtained as follows:

(12) $$\begin{array}{c} k_{1}=0.0261,\;\;k_{2}=0.0142,\;\;k_{3}=1.0459. \end{array}$$

Then the sediment carrying capacity formula can be written as follows:

(13) $$\begin{array}{c} S_{*}=0.0261\frac{U^{2}D_{50}}{gh^{2}}+0.0142\frac{U^{3}}{gh\omega }+1.0459, \end{array}$$

where *S*_∗_ is the sediment carrying capacity (kg/m^3^); *U* is average flow velocity (m/s); *D*_50_ is the median grain size (mm); *h* is the average water depth (m); *ω* is the average settling velocity (mm/s).

Then, (13) is the formula of the sediment carrying capacity.

## 3. Verification and Comparison

The measured data covers several types of rivers at home and abroad. In the paper, we will adopt 2633 measured data to fit and verify the derived formula. The parameter values of 2633 measured data in different rivers are shown in Table 1.

These natural rivers include the Niobrara River (see [20]), the Atchafalaya River (see [21]), the Rio Grande River (see [22]), the Missouri River (see [23]), the Sacramento River (see [24]) in USA, the North Saskatchewan River and the Elbow River in Canada (see [25]), Columbian River (see [26]), and some rivers in India and Pakistan [27, 28, 39].

In 1955, Cobly and Hembree observed the Niobrara River near Lusk and won the measured data of flow velocity, median grain size, water depth, sediment concentration, and so on. Niobrara River is 720 km long and the basin area is 31,080 km^2^. In 1968, Toffaleti observed the Atchafalaya River, a tributary of the Red River, and the Mississippi River and got the measured data. From the observed results it can be found that the Atchafalaya River is 360 km long, the range of flow velocity is 0.21 to 3.56 m/s, and the range of water depth is 4.163 to 13.28 m; this observed data had higher reliability. In 1972, Culbertson et al. observed the Rio Grande River, one of the largest rivers in North America; it is about 3,033 km long and obtained some valuable measured data. In 1978, Shen et al. observed the Missouri River and got some measured data. From the observed results it can be found that this river is over 4300 km long, the range of flow velocity is 1.389 to 1.745 m/s, and the range of water depth is 2.714 to 4.238 m. In 1990, Nakato observed the Sacramento River, the longest river in California, and obtained some useful measured data. From the observed results it can be seen that the river is 614.8 km long and the ranges of flow velocity, water depth, and median grain size are wide; they are suitable for fitting formula.

**Figure 1:**
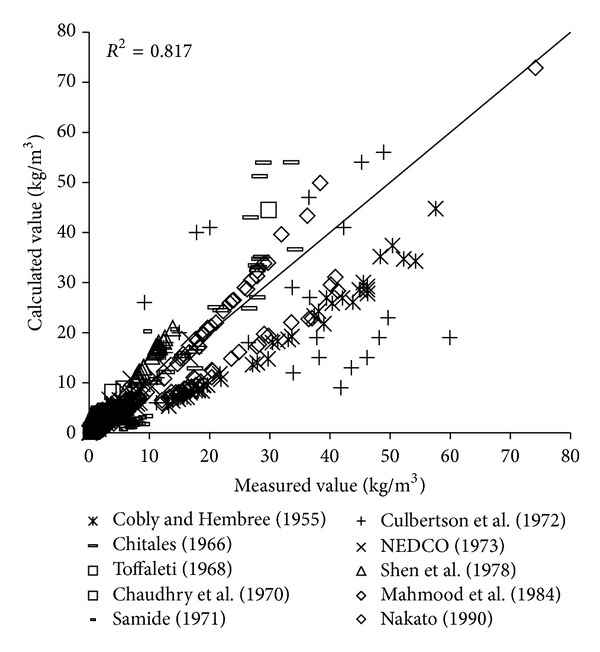
Verification diagram of Cao Formula.

**Figure 2:**
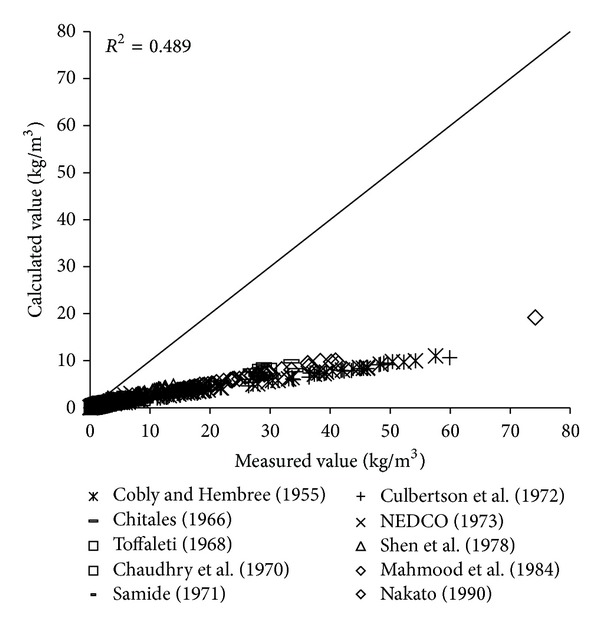
Verification diagram of Li Formula.

**Figure 3:**
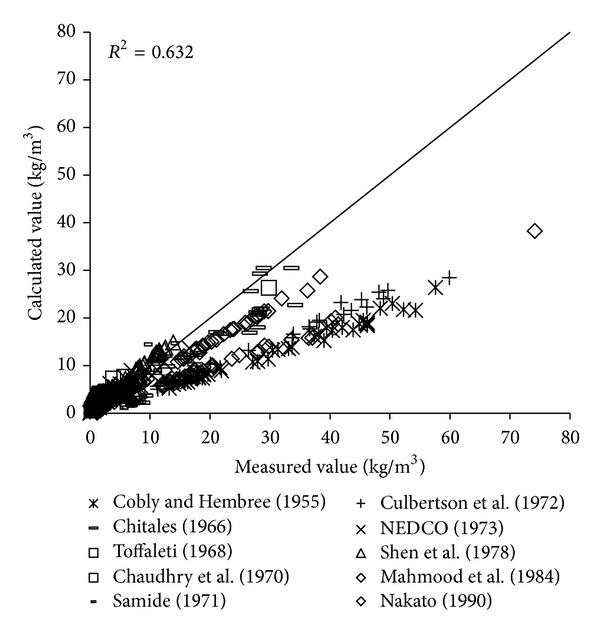
Verification diagram of Zhang Formula.

**Figure 4:**
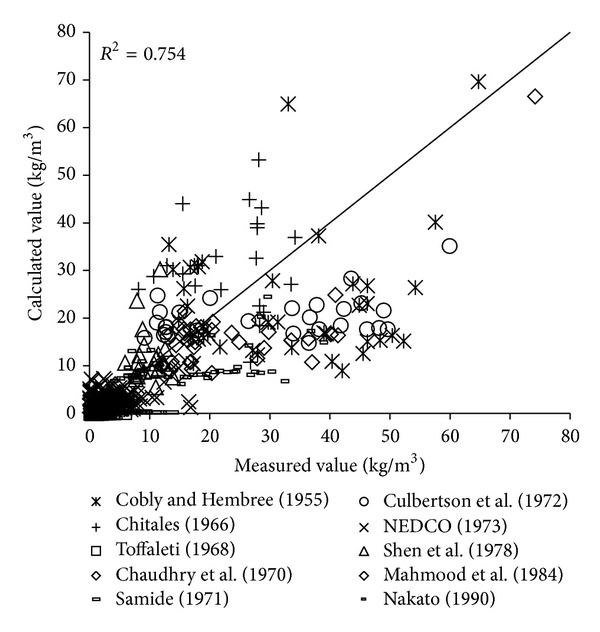
Verification diagram of A.-W. Formula.

**Figure 5:**
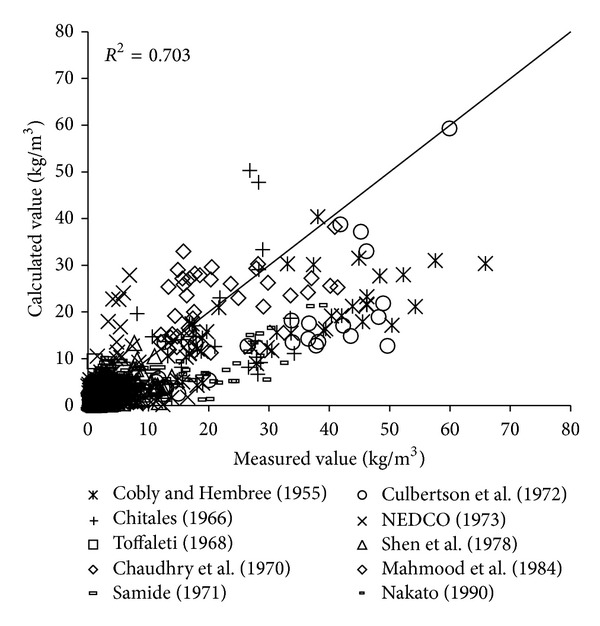
Verification diagram of E.-H. Formula.

**Figure 6:**
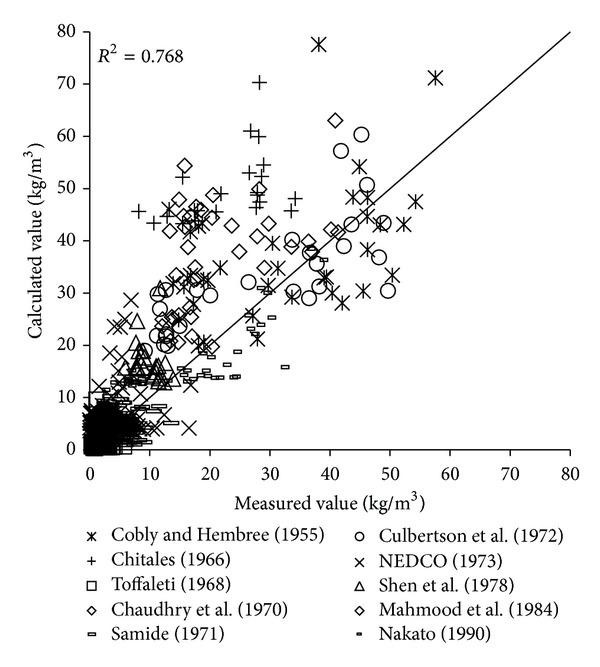
Verification diagram of Yang Formula.

**Figure 7:**
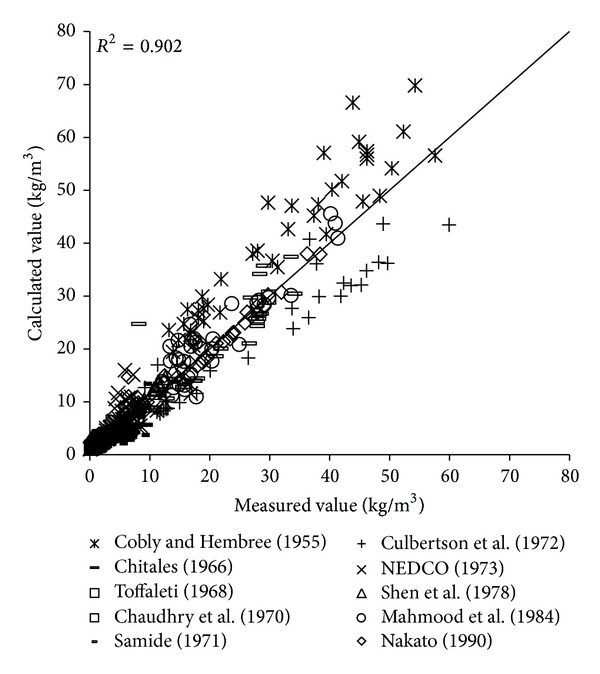
Verification diagram of derived formula. A.-W. Formula represents Ackers-White Formula; E.-H. Formula represents Engelund-Hansen Formula.

To verify the reliability of the derived formula, the paper will use the remaining 1000 measured data to verify and analyze the derived formula. The results of verification of all formulas can be seen in Figures 1, 2, 3, 4, 5, 6, and 7.

In the figures, *x*-axis is the measured value and *y*-axis is the calculated value.

According to the verified results it can be seen that the fitting degree of derived formula is higher than other formulas. Cao Formula and Yang Formula are better. Also, combined with correlation coefficients and theories of all formulas it can be found that the formula taking into account the median grain size in calculation has high precision. Therefore, we need to take further verification of the derived formula whether the median grain size is a main factor influencing the sediment carrying capacity.

In order to further verify the derived formula, the paper will use the results of flume tests of Gilbert [30], Brownlie [40], Einstein [41], Einstein-Chien [31], Brooks [32], Guy et al. [33], Song et al. [34], and Wang et al. [35]. The parameter values of measured data in the flume tests by the scholars are shown in Table 2.

By taking into account the change in difference range of median grain size, Gilbert (1914) had done several flume tests in different size of flumes; the range of median grain size is 0.305~7.01, and the range of different size flume is 0.2012~0.5974. Similar to Gilbert (1914), Meyer-Peter-Muller [42] had done three sets of tests, respectively: when the range of median grain size is 0.52 to 5.2 mm, the corresponding flume width is 0.3539 m; when the median grain size is 3.3 mm, the corresponding flume width is 0.4999 m; when the range of median grain size is 0.38 to 28.65 mm, the corresponding flume width is 1.9998 m. The flume width in Einstein (1950) flume tests is 0.2667 m, and the range of corresponding median grain size is 0.11 to 0.91 mm. With Chien, Einstein had done other flume tests in 1953; the flume width is 0.3048 m, and the range of corresponding sediment grain size is 0.095 to 0.385 mm. The flume width in Brooks [32] flume tests is 0.2667 m, and the range of corresponding sediment grain size is 0.088~0.145 mm. Guy et al. [33] had done two sets of flume tests in different flume: when the range of median grain size is 0.32 to 0.59 mm, the corresponding flume width is 0.6096 m; when the median grain size is 0.19~1.20 mm, the corresponding flume width is 2.4384 m. The flume width in Song et al. (1998) flume tests is 0.5998 m, and the corresponding median grain size is 12.3 mm. The flume width in Wang et al. (1998) flume tests is 0.5 m, and the corresponding median grain size is 0.18 to 13 mm.

**Figure 8:**
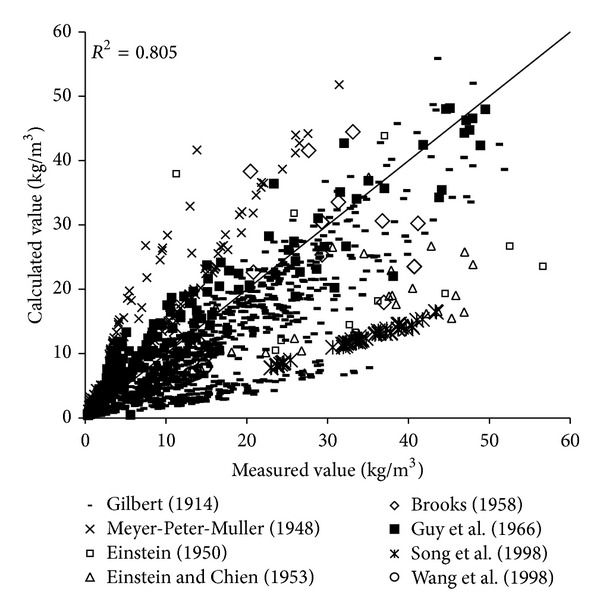
Verification diagram of Cao Formula.

**Figure 9:**
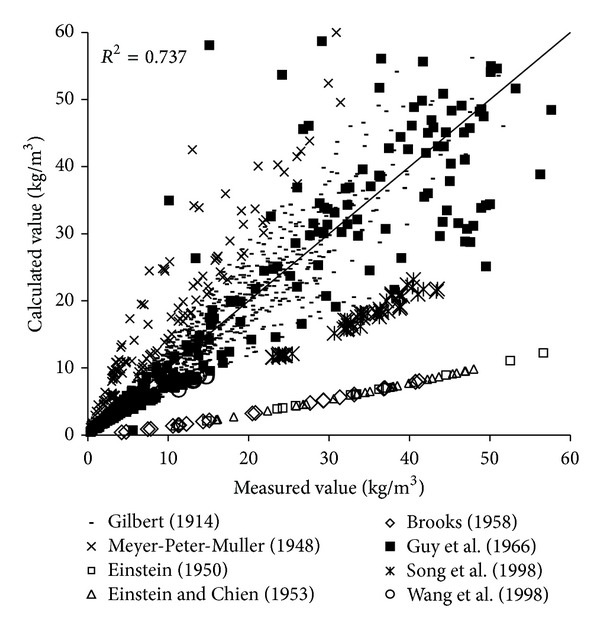
Verification diagram of Li Formula.

**Figure 10:**
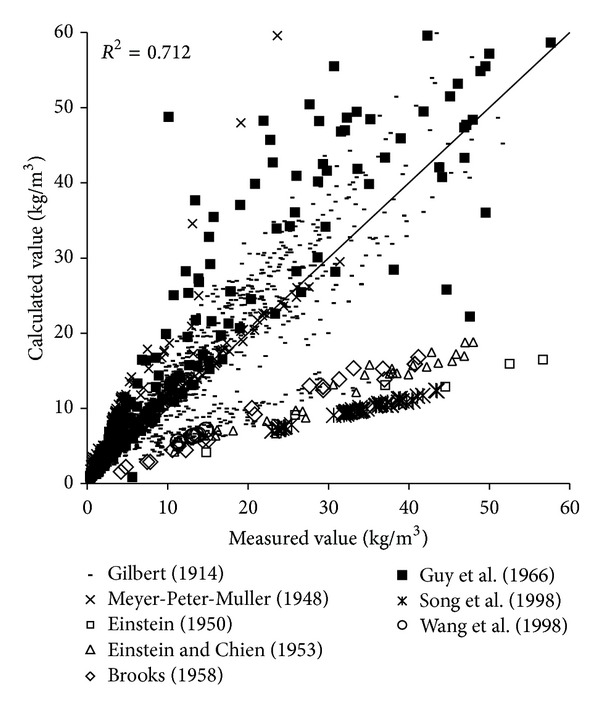
Verification diagram of Zhang Formula.

**Figure 11:**
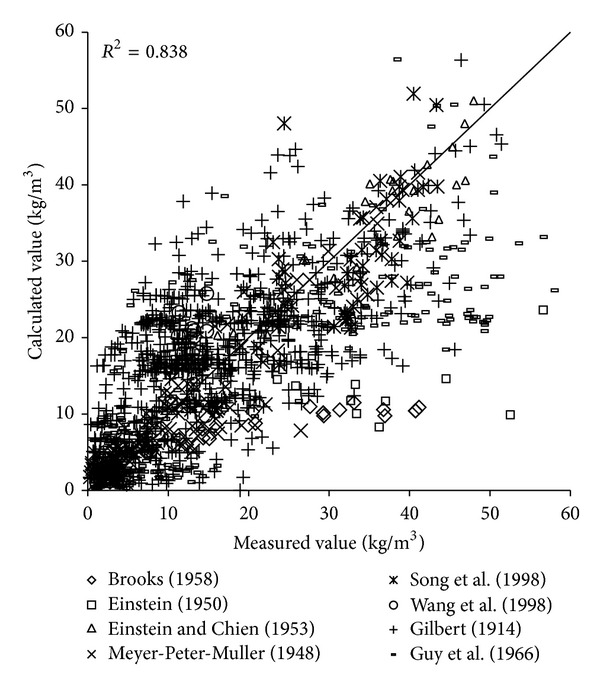
Verification diagram of A.-W. Formula.

**Figure 12:**
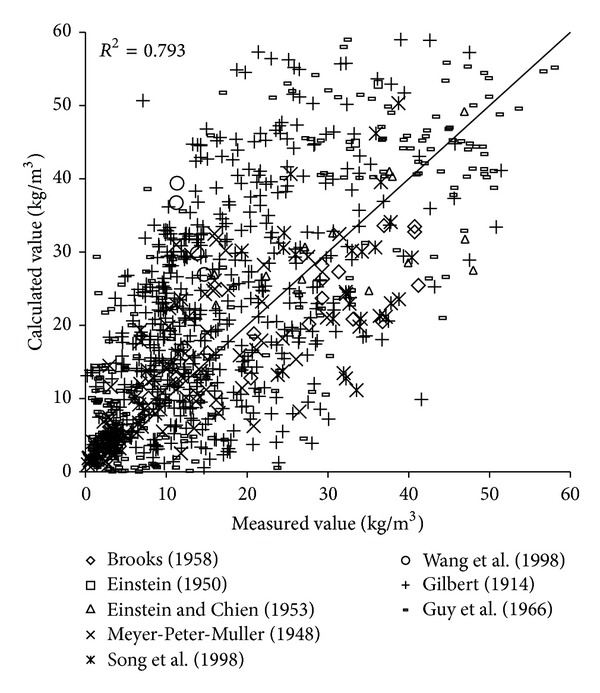
Verification diagram of E.-H. Formula.

**Figure 13:**
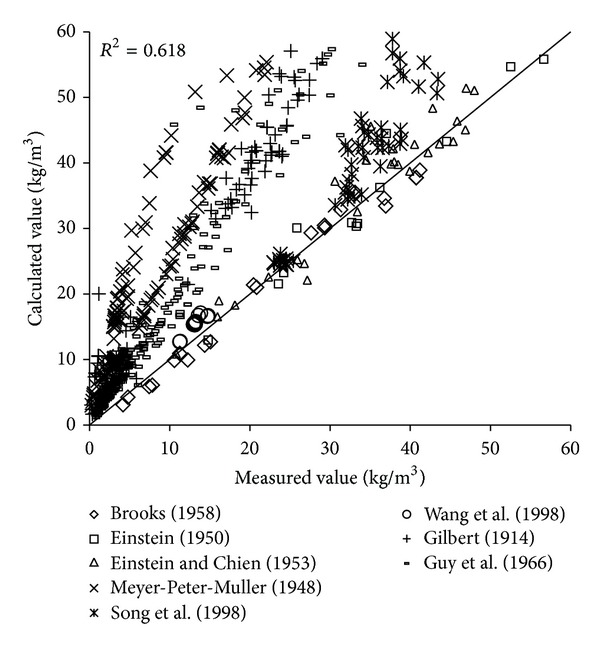
Verification diagram of Yang Formula.

**Figure 14:**
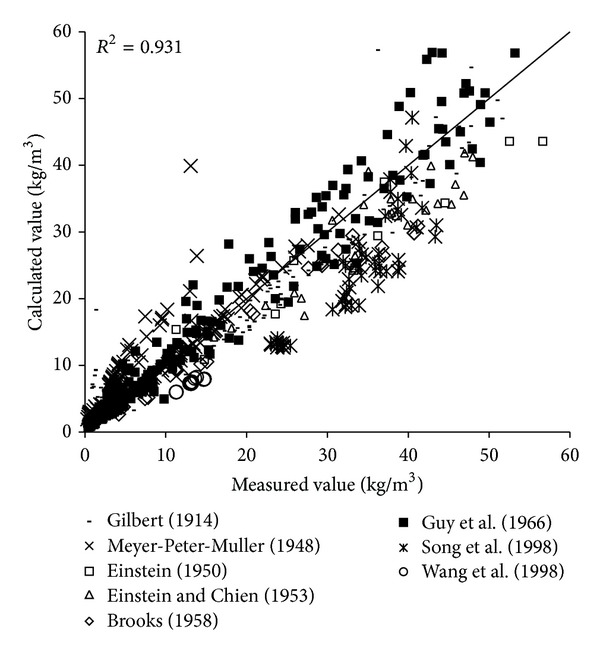
Verification diagram of derived formula.

The results of verification of all formulas can be seen in Figures 8, 9, 10, 11, 12, 13, and 14.

In the figures, *X*-axis is the measured value and *Y*-axis is the calculated value. According to the verified results of the flume tests it can be found that the result of derived formula is the closest to the measured data. The further verification illustrated the rationality of the derived formula, and it can be a reference in engineering.

## 4. Error Analysis

According to the verified results, the errors by the formulas are shown in Tables 3 and 4.

From the tables it can be found that the error by derived formula is the least compared to the other six formulas. When verifying with the measured data of natural rivers, the average error is 4.05%; when verifying with the measured data of flume tests, the average error is 4.57%. If calculated by other formulas, the average errors are in the range of 7.16%~37.66% and 6.88%~13.66%, respectively. Taking into account the scale effect of flume tests, it is understandable that the error by flume tests is bigger than that by natural rivers data. From the error analysis it can be seen that it is feasible to take the median grain size as one main influencing factor.

Meanwhile, according to the error analysis data it can be also found that the percentage of scatters in which the error is less than 10% and less than 20% verified by derived formula are less than those by the others formulas, respectively, are 88%, 96% and 85%, 92%.

In conclusion, the derived formula is simple in principle, practical in implementation, and reasonable in results, and it further illustrates that the view of considering the median grain size in determining the sediment carrying capacity is correct. This view is consistent with Chien.

## 5. Conclusions

According to the present analysis and study, some conclusions can be drawn as follows.

1. By taking into account the influence of median grain size on the sediment carrying, a new sediment carrying capacity formula combined with the turbulence intensity of the flow (*U* ^2^/*gh*), gravity action (*U*/*ω*), and relative roughness (*D*_50_/*h*) is established.
2. From the verified results it can be seen that the derived formula has high accuracy by using the method of dimensional analysis and combined with multivariate analysis and linear least squares method, it is a strong theoretical and practical method.
3. The results further verified by flume tests showed that the precision by introducing the relative roughness in deriving formula is higher than other formulas. It means that taking into account the median grain size in sediment deposition is reasonable and feasible.

## Figures and Tables

**Table 1:** The parameter value of each river.

Number	Source	*V* (m/s)	*H* (m)	*D*_50_ (mm)	*ω* (mm/s)	*S*_∗_ (kg/m^3^)	Amount
1	Cobly and Hembree (1955) [20]	0.63~1.71	0.191~0.481	0.206~1.359	1.168~2.447	23.22~47.6	40
2	Toffaleti (1968) [21]	0.21~3.56	4.163~13.28	0.085~0.129	1.07~1.265	0.127~13.994	305
3	Culbertson et al. (1972) [22]	0.59~1.618	0.193~1.712	0.170~0.250	1.594~2.192	39.178~59.95	33
4	Shen et al. (1978) [23]	1.389~1.745	2.714~4.238	0.113~0.166	1.258~2.113	5.117~13.90	25
5	Nakato (1990) [24]	0.124~1.693	1.012~6.163	0.1350~4.300	1.312~3.522	1.245~7.107	29
6	Samide (1971) [25]	1.183~3.622	0.752~2.243	13.61~57.51	1.543~3.163	30.52~78.78	55
7	NEDCO (1973) [26]	0.117~1.389	0.32~11.228	0.100~1.080	1.155~4.358	0.029~6.86	113
8	Chitales (1966) [27]	0.138~1.954	0.271~3.156	0.021~0.052	0.117~1.643	12.4~18.61	32
9	Chaudhry et al. (1970) [28]	0.631~1.197	1.911~3.114	0.190~0.311	1.613~2.297	2.185~8.26	33
10	Mahmood et al. (1984) [29]	0.092~2.128	0.113~11.75	0.162~0.579	0.843~2.898	0.115~22.44	1968

**Table 2:** The parameter value of each flume test.

Number	Source	*V* (m/s)	*H* (m)	*D*_50_ (mm)	*ω* (mm/s)	*S*_∗_ (kg/m^3^)	Amount
1	Gilbert (1914) [30]	0.23~1.55	0.011~0.224	0.305~7.01	2.273~11.113	12.04~63.95	889
2	Meyer-Peter-Muller (1948) [42]	0.23~2.88	0.008~1.092	0.38~28.65	2.551~22.47	0.6~41.08	140
3	Einstein (1950)	0.54~1.41	0.099~0.139	0.11~0.91	1.233~3.242	108.58~435.84	30
4	Einstein and Chien (1953) [31]	0.65~1.097	0.177~0.237	0.095~0.385	1.155~2.569	142.29~412.5	26
5	Brooks (1958) [32]	0.25~0.66	0.047~0.091	0.088~0.145	1.096~1.505	26.84~308.53	23
6	Guy et al. (1966) [33]	0.198~1.932	0.058~0.405	0.19~1.20	1.758~4.587	0.348~145.14	339
7	Song et al. (1998) [34]	0.9~1.18	0.084~0.253	12.3	14.723	22.98~43.48	54
8	Wang et al. (1998) [35]	0.6~1.43	0.035~0.155	0.18~13	1.705~15.14	11.33~89.90	33

**Table 3:** The results of the error calculated by each formula in the natural rivers.

Formula	*E*_10%_ (%)	*E*_20%_ (%)	Average error (%)
Cao Formula	76%	88%	7.16%
Li Formula	31%	39%	37.66%
Zhang Formula	62%	73%	12.56%
A.-W. Formula	71%	82%	9.33%
E.-H. Formula	68%	79%	10.02%
Yang Formula	73%	85%	8.91%
Derived formula	88%	96%	4.05%

*E*_10%_ represents the percentage of scatters in which the error is less than 10% of measured data in the natural rivers; *E*_20%_ represents the percentage of scatters in which the error is less than 20% of measured data in the natural rivers.

**Table 4:** The results of the error calculated by each formula in the flume tests.

Formula	*E*_10%_ (%)	*E*_20%_ (%)	Average error (%)
Cao Formula	78%	90%	8.76%
Li Formula	69%	80%	9.86%
Zhang Formula	73%	83%	10.06%
A.-W. Formula	81%	90%	6.88%
E.-H. Formula	76%	88%	9.13%
Yang Formula	61%	70%	13.66%
Derived formula	85%	92%	4.57%

*E*_10%_ represents the percentage of scatters in which the error is less than 10% of measured data in the natural rivers; *E*_20%_ represents the percentage of scatters in which the error is less than 20% of measured data in the natural rivers.
